# Mating opportunities in *Sangalopsis veliterna* females: Costs and benefits

**DOI:** 10.1093/jis/14.1.112

**Published:** 2014-08-12

**Authors:** Linda C. Hernández Duran, Gonzalo E. Fajardo Medina, Luz S. Fuentes Quinter, Oliver Martin

**Affiliations:** 1 Departamento de entomología, Centro de Bio-Sistemas Universidad Jorge Tadeo Lozano; 2 Facultad de ciencias biológicas e ingeniería, Universidad Jorge Tadeo Lozano

**Keywords:** mating rate, fitness, longevity, fecundity, fertility

## Abstract

In nature, females of several animal taxa exhibit considerable variation in their mating system, and this variation involves different balances of costs (e.g., energetic, reproductive) and benefits (e.g., increased net reproductive rate of the female, increased longevity). Many studies have focused on discovering the potential advantages and disadvantages that females could have when increasing their mating rate and the possible evolutionary consequences that may result. Butterflies and moths are an ideal study system because it is easy to determine and to manipulate experimentally their mating frequency. In this study, the effect of continuous availability of different numbers of males (1, 2, 4, 8) on female mating rate and fitness components was estimated by comparing the number of spermatophores in the corpus bursa (an estimate of the number of copulations, but not of the number males involved in these copulations), female longevity, lifetime number of laid eggs (fecundity), and proportion of hatching eggs (fertility) in the moth *Sangalopsis veliterna* Druce (Lepidoptera: Geometridae). The results showed that there were no significant differences in either fertility or fecundity when treatments were compared, but longevity and in some cases fecundity increased when females had several matings.

Resumen

En la naturaleza, hembras de varios taxa animal muestran una variación considerable en su sistema de apareamiento, esta variación involucra diferentes costos (energéticos, reproductivos, etc) y beneficios (aumento de la tasa reproductiva neta de la hembra, mayor longevidad, entre otros). En años recientes, muchos estudios se han enfocado en descubrir las potenciales ventajas y desventajas que las hembras podrían tener al aumentar su número de cópulas y las posibles consecuencias evolutivas que podrían resultar. Las mariposas y polillas son un sistema de estudio ideal, dada la facilidad para determinar y manipular experimentalmente su frecuencia de apareamiento. En este estudio, el efecto de la disponibilidad continua de diferente número de machos (1, 2, 4, 8) sobre la frecuencia de apareamiento de las hembras y los componentes del potencial reproductivo (fitness) fueron estimados al comparar el número de espermatóforos hallados en la bursa copulatrix (un estimado del número de copulas, pero no del número de machos involucrados en estas copulas), la longevidad de las hembras, número de huevos colocados (fecundidad) y proporción de huevos fecundados (fertilidad) en la polilla *Sangalopsis veliterna* Druce (Lepidóptera: Geometridae). Los resultados indican que no existen diferencias en la fertilidad ni en la fecundidad; sin embargo la longevidad aumentó a medida que se las hembras presentaron más de una cópula y de igual manera en algunos casos la fecundidad aumentó con varios apareamientos.

## Introduction

Under natural conditions, females of many animal taxa maximize their reproductive success by mating multiple times with different males ( Wedell 2001 , Stockley 2003 , Nilsson 2004 ). Nevertheless, there is a considerable variability in the frequency of matings, ranging from strictly monandrous species or individuals (females mate only with one male in a breeding season) to polyandrous species or individuals (females mate with multiple males in a breeding season) ( Wiklund et al. 1993 , Wedell et al. 2002 ). Because polyandry seems to be the most common reproductive strategy in insects, especially in Lepidoptera ( Arnqvist and Nilsson 2000 ), several studies have focused on the adaptative value of this reproductive pattern ( Arnqvist and Nilsson 2000 , Wedell et al. 2002 , Nilsson 2004 , Gowaty et al. 2010 ).

Independent of the reproductive habits shown by females, mating is a process that generates costs ( Arnqvist 1989 , Chapman et al. 1995 , Nilsson 2004 ). These include high energy consumption, predation risk, and mobility reduction, among others ( Arnqvist 1989 ). Several hypotheses attempt to explain why females might benefit from multiple matings, for example, increased fecundity, fertility, longevity, and greater genetic diversity in their offspring ( Arnqvist and Nilsson 2000 . Wedell et al. 2002 , Nilsson 2004 , Harano et al. 2006 , Gowaty et al. 2010 ). It also is worth noting that the selective forces on males and females to maximize their reproductive success rarely coincide, probably due to factors such as the difference in energy investment in gamete production, parental care, and ways to achieve reproductive success ( Wiklund et al. 2001 , Hosken and Snook 2005 , Wedell 2005 ).

Studies of insects have expanded our understanding of the mating patterns of animals and especially the role of females in the scenario of sexual selection before and after copulation ( Arnqvist and Rowe 2002 ). Butterflies and moths are an ideal study system because it is relatively easy to determine their mating frequency by counting spermatophore remains retained in the reproductive tract of females, which stay in place throughout her life ( Wedell 2005 ). Males generally transfer only one spermatophore per mating ( Drummond 1984 ). In several studies, counting spermatophores has made it possible to test predictions about mating frequency, optimal reproductive rates, and ecological costs and benefits associated with fitness in natural conditions and even in captivity.

In this study, we used the moth *Sangalopsis veliterna* Druce (Lepidoptera: Geometridae) as a model to evaluate predictions about mating frequency according to males’ availability and their possible effects over their fitness. *S. veliterna* is a forest pest species in Colombia and is well suited for ecological studies due to its short lifespan and ease of breeding in the laboratory. The aims of this study were (i) to determine the mating pattern of *S. veliterna* females under natural conditions, (ii) to assess the effect of continuous availability of different numbers of males on female mating rate (mating frequency), and (iii) to assess the effect of mating frequency on female fitness components. To achieve this, the number of spermatophores found in the corpus bursa of field-collected females were used to determine the mating pattern of *S. veliterna;* the spermatophore count obtained from experimental females was used to measure the effect of availability of different number of males on female mating rate (an estimate of the number of copulations, but not males involved in these copulations); also, female longevity, lifetime number of laid eggs (fecundity), and the proportion of hatching eggs (fertility) of females exposed throughout their lifetime to different numbers of males were measured.

## Materials and Methods

### Breeding design

Individuals used in this study were obtained from a culture established at the laboratory of the Centro de Biosistemas at the Jorge Tadeo Lozano University (Chia, Colombia), at an average temperature of 23.1 ± 2.0°C, 75% RH, and 12:12 L:D. Adults were fed with pollen of castor oil plant *(Ricinus* spp. (Malpighiales: Euphorbiaceae)) and a 10% honey solution; leaves of *Croton* spp. (Malpighiales: Euphorbiaceae) trees were used for oviposition and subsequent larval feeding.

### Determining the mating pattern of *S. veliterna* females

To determine the mating pattern of *S. veliterna,* 30 wild females collected in the city of Bogotá were dissected, and the spermatophores found inside their reproductive tract were quantified. For the specimens derived from culture, wings were marked to distinguish male from female individuals. Daily observations were made to establish the number of copulations per female (mating rate) and thereby determine the mating pattern of *S. veliterna* females.

### Assessment of mating frequency and fitness components of *S. veliterna* females

To assess the effect that a different offer of males could have on mating frequency and fitness components (longevity, fecundity, and fertility) of *S. veliterna* females, females were exposed to different numbers of males as follows: TP: 15 virgin females were held with 15 virgin males, promiscuous (unrestricted) mating; T1: virgin females were held with a single male over their entire life and exposed to repeated mating (19:1(5*); virgin females were allowed constant access to varying number of males (2, 4, 8), as follows: T2: 19:2(5*, T3: 19:4(5*, and T4: 19:8(5*. At the beginning, all treatments were compared to each other (see statistical analysis). Then, T1 (19:1d*) was compared with T2, T3, and T4 (19:2(5*, 19:4(5* and 19:8(5*); these latter were pooled, considering that all of them follow polyandry criteria (females exposed to more than one male and with multiple matings). Similarly, previously the variables T2, T3, and T4 were compared to each other, and no differences were observed; this made it possible to compare T1 to females with more than one male (T2, T3, T4). These analyses were carried out taking into account the number of spermatophores to assess mating pattern and three fitness components measured in this study (longevity, fecundity, and fertility).

For each experiment, 15 replicates were carried out for 20 days. The moth groups were kept in plastic 500 mL vials. For feeding, cotton balls soaked with a solution of 10% honey were provided to adults in each chamber. Every day the number of laid eggs (fecundity) and hatched larvae (fertility) were recorded. After the females died, their age (in days) and the number of spermatophore remains present in the female reproductive tract were recorded under a stereomicroscope (Olympus SZ61, www.olympus-global.com ).

### Statistical analysis

When the normality of the variables was tested using the Shapiro-Wilk test, it was found that these were not distributed normally; therefore nonparametric Kruskal-Wallis tests were carried out to evaluate differences between treatments (TP, T _1_ , T _2_ , T _3_ , T _4_ ), followed by a post-hoc Dunn's test. After that, to compare mating pattern and fitness components among females exposed to one male (T _1_ : 1♀:1♂) to to females that mated with more than one male (T _2_ , T _3_ , T _4_ ), the variables T _2_ , T _3_ , and T _4_ were compared and subsequently pooled and compared with T _1_ . To evaluate the possible differences among these variables, a Mann Whitney *U* test was carried out *.* All analyses were performed using the Statistica V.10 software (Statsoft, www.statsoft.com ). The significance level was set to <0.05.

## Results

### Mating pattern of *S. veliterna* females

**Figure 1.**
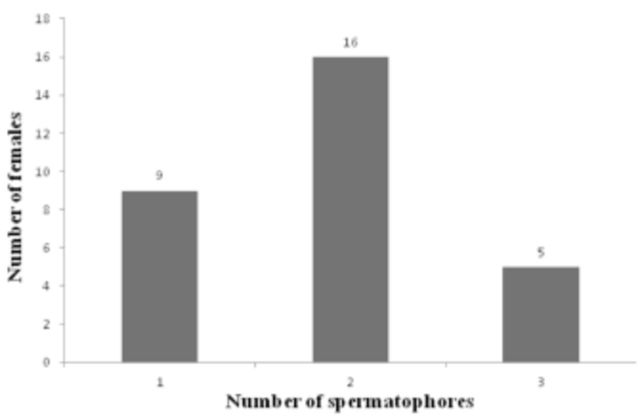
Number of spermatophores per dissected wild *Sangalopsis veliterna* female.

In this study, 30 wild females had an average of 1.9 ± 0.7 spermatophores per female; 53% presented more than one spermatophore, and 16.6% presented more than three spermatophores in their reproductive tract ( Fig. 1 ). From this, it was established that they mated repeatedly throughout their reproductive cycle, confirming a polyandrous mating pattern.

### Mating frequency

**Figure 2.**
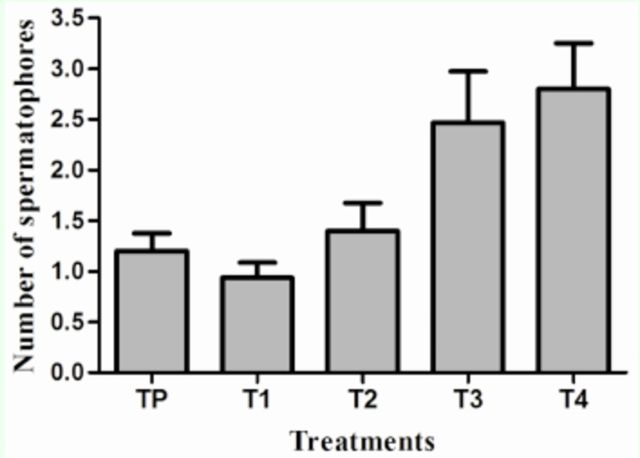
Comparison of *Sangalopsis veliterna* females’ mating under different treatments. TP: 15 virgin females were held with 15 virgin males; T1: virgin females were held with a single male over their entire lives; T2: 1 **$** :2 **(5\** T3: 1 **$** :4 **^** and T4: 1 **$** :8 **(5\** virgin females were allowed constant access to different number of males (Kruskal Wallis H1, 14 = 12.95, *P* = 0.0047).

**Figure 3.**
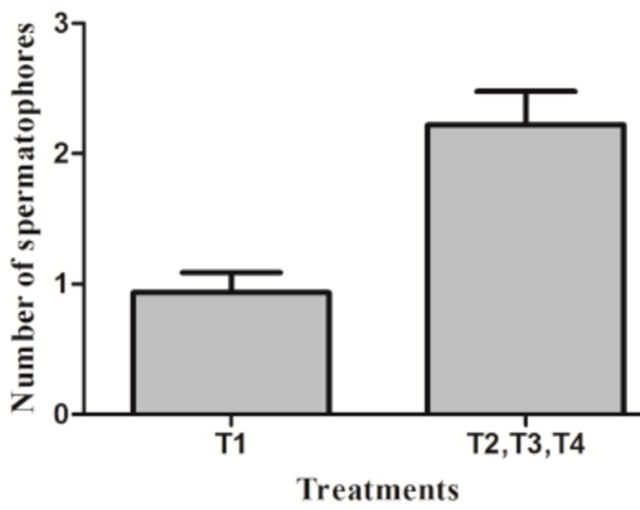
Comparison of *Sangalopsis veliterna* female mating frequency between T1 (T1: 1 **$** :1 **c?** ) and females with more than one male (T2, T3, T4). Mann-Whitney *U:* 177.0, *P* = 0.0047.

Females without mating restriction (TP) and exposed to a single male (T _1_ ) showed between one and two spermatophores. When offered multiple potential mating partners (T _2_ , T _3_ , T _4_ ), an increase in the number of matings was observed; this finding indicates that mating frequency of *S. veliterna* is affected by the number of available males per female **(**Fig. 2 ; Kruskal Wallis H _1,__14_ = 12.95, *P* = 0.0047). Similarly, when T _1_ was compared with T _2_ , T _3_ , and T _4_ , the number of spermatophores increased with availability of males ( Fig. 3 ; Mann-Whitney *U* : 177.0, *P* = 0.0047).

### Fitness components of *S. veliterna* females

**Figure 4.**
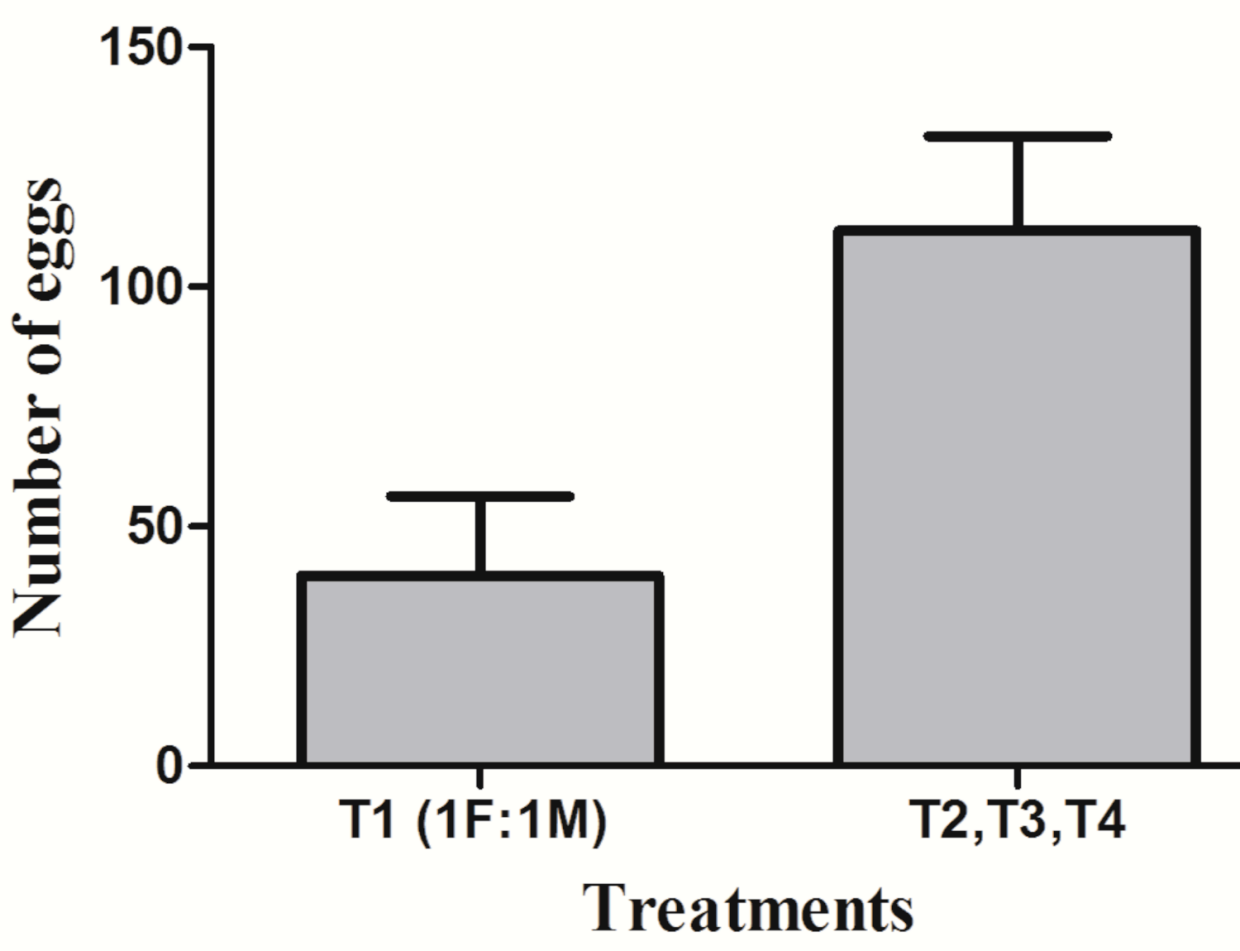
Fecundity of *Sangalopsis veliterna* females comparing T1 (1 **$** :1 **(3\** repeated mating) to females with more than one male (T2, T3, T4). Mann-Whitney test *U:* 223.5, *P* = 0.0245.

Regarding fecundity (number of laid eggs) (Kruskal-Wallis; H _1,__14_ = 4.480, *P* = 0.2141) and fertility (number of hatched larvae) (Kruskal-Wallis; H _1,__14_ = 3.602, *P* = 0.3173), no significant difference was observed between treatments. These results match the results obtained in the studies carried out by Tregenza and Wedell (1998) and Rincón and García (2007) .When T _1_ (1♀:1♂) was compared to females that had constant access to more than one male (T _2_ , T _3_ , T _4_ ), significant differences were observed in fecundity ( Fig. 4 ; Mann-Whitney U: 223.5, *P* = 0.0245) but not in fertility (Mann-Whitney *U* : 47.50, *P* = 0.2442).

**Figure 5.**
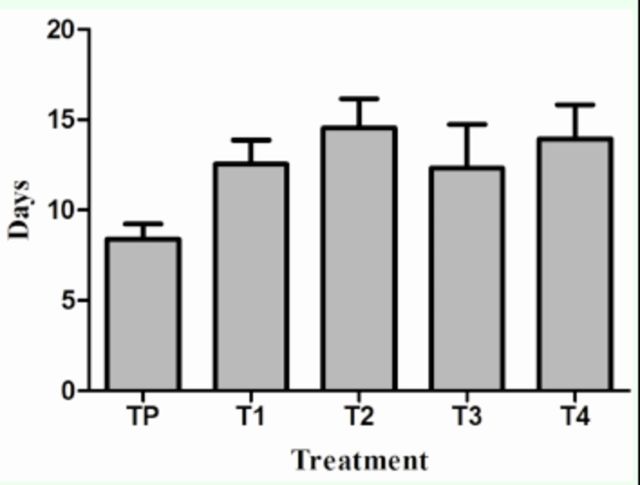
Female longevity *Sangalopsis veliterna* (days). Comparison between treatments (Kruskal-Wallis; H1, 14 = 9. 530, *P* = 0.0453).

When longevity was compared between treatments, the Kruskal-Wallis test detected differences in longevity as a function of treatments ( Fig. 5 ; Kruskal-Wallis; H _1,__14_ = 9. 530, *P* = 0.0453), which indicates that females that have a different availability of males and mate more than once (T _2_ , T3, and T _4_ ) live longer than TP females. When T _1_ was compared to T _2_ , T _3_ , and T _4_ , no significant differences were observed in longevity, indicating that females with different availability of males (T _1_ , T _2_ , T _3_ , T _4_ ) do not differ in their longevity (Mann-Whitney *U* : 337.0, *P* = 0.5000).

## Discussion

### Mating pattern of *S. veliterna*

As Parker (1979) , Drummond (1984) , and Simmons (2001) observed, polyandry is a reproductive strategy in which females of different taxa mate with more than one male throughout their life. Polyandry is very common in the Lepidoptera order, although it has been observed that sometimes females of species with polyandrous mating behavior in the laboratory tend to mate only once in wild ( Wedell 2001 ). The results in this study showed that *S. veliterna* females presented more than one spermatophore in their reproductive tract, which indicates a polyandrous mating pattern. Likewise, we observed that 30% of wild females had a single spermatophore and 70% of females with multiple males availability had more than two spermatophores ( Fig. 1 ). It is important to highlight that in this study the number of spermatophores inside the reproductive tract was quantified, but it was not determined whether these spermatophores came from the same male or different males.

### Costs and benefits associated with mating frequency and fitness components.

The results showed a clear relationship between number of available males and the frequency of female matings ( Figs. 2 and 3 ). However, because of the naturally observed sex ratio in the population of *S. veliterna* (i.e., three females per two males; Hernández 2011 ), mating frequency of females could be determined by sex ratio and probability of meeting. This could decrease the opportunity for multiple matings and the potential benefits on the fitness of females ( Tregenza and Wedell 1998 , Simmons 2001 , Wiklund et al. 2001 , Chapman et al 2003 , Nilsson 2004 , Jones and Ratterman 2009 ). It also should be taken into account that in the treatments that had a greater availability of males (T _2_ , T _3_ , and T _4_ , either when comparing among treatments or when comparing pooled data), females could accept further matings (probably decreasing costs associated with mating) and thereby avoid harassment from males, as males could cause physical (wings, legs, etc) and reproductive damage when trying to mate ( Parker 1979 , Thornhill and Alcock 1983 , Simmons 2001 , Wiklund et al. 2001 , Nilsson 2004 , Hosken and Snook 2005 , Harano et al. 2006 ).

In the case of females of *S. veliterna* , no significant differences in terms of fecundity were observed when different treatments were compared (Kruskal-Wallis; H _1,__14_ = 4.480, *P* = 0.2141). However, when T _1_ was compared with T _2_ , T _3_ , and T _4_ (not including TP, because this treatment had no mating restriction and there was more than one female), females that were held with a single male through their entire life cycle and presented repeated matings had less fecundity than T _2_ , T _3_ , and T _4_ females ( Fig. 4 ; Mann-Whitney U: 223.5, *P* = 0.0245). Females that mate with more than one male possibly could benefit from donations made by the males by having an increased reproductive rate (through the processing of nutrients from males at the time of mating) and possible somatic maintenance ( Wiklund et al. 2001 , Hosken and Stockley 2003 , Nilsson 2004 , Torres-Villa and Jennions 2004 , Arnqvist and Andres 2006 ).

In butterflies and moths, male seminal compounds may contain nutrients, anti-aphrodisiacs, and gonadotropic hormones, which can have a positive, negative, or neutral impact on the fitness of females ( Boggs and Gilbert 1979 , Gwynne 1984 , Arnqvist and Nilsson 2000 ; Simmons 2001 , Wiklund et al 2001 , Wedell et al. 2002 ; Hosken and Stockley 2003 , Nilsson 2004 , Mcnamara et al. 2008 ). In many species, it has been observed that females who mate multiple times (polyandrous) may receive benefits from the ejaculates of males either in terms of fecundity, egg size, or longevity ( Arnqvist and Nilsson 2000 , Wedell et al. 2002 , Nilsson 2004 , Arnqvist and Andres 2006 , Mcnamara et al. 2008 ).

Fertility was not affected by the number of copulations in this study. However, it is probable that one or two matings would be enough to fertilize all the eggs laid by a female throughout her reproductive cycle, and a greater number of copulations could mean a use of these resources for somatic maintenance but not for their offspring. It would be important, however, to determine whether it is possible that the benefits of multiple matings with different males are reflected in an indirect way (genetically, by increasing genetic diversity and reproductive success of their offspring) in the offspring of females who were exposed to a greater number of copulations ( Thornhill and Alcock 1983 , Tregenza and Wedell 1988, Jennions and Petrie 2000 , Nilsson 2004 ). Similarly, it must be taken into account that females of this study were fed with a diet rich in carbohydrates (10% honey solution), so it is possible that the benefits from the spermatophores of males only contributed in a minor proportion to the energy budget of females. Besides, the positive energetic effects of polyandry might be more evident when experiments with diet restrictions are carried out, as described by Arnqvist and Nilsson (2000) .

In Lepidoptera, male seminal products boost the survival and egg production of females ( Boggs and Gilbert 1979 , Gwynne 1984 , Cordero 1995 , Simmons 2001 , Wedell et al 2002 , Välimäki et al 2006 , Mcnamara et al 2008 ). This might be the case for *S. veliterna* females, which differed significantly in their longevity (when all treatments were compared) and egg production (only when T1 was compared to T2, T3, T4), but not in fertility ( Figs. 4 and 5 ); they could invest male nutritional substances for their own survival and increase the production of eggs, as observed in the study conducted by Mcnamara et al. (2008) . It is worth noting that females without mating restriction (TP) showed a decrease in longevity in contrast to females that mated more than once or had mating restrictions ( Fig. 5 ). This could be explained because TP females probably suffered greater harassment from males, as well as more intense competition between females for space and resource. It would be interesting to investigate whether females in the wild that do not have the opportunity to mate with more than one male suffer a reduction in their longevity.

Situations similar to this study could be found in nature, where matings are determined by many ecological and behavioral variables, and depending on the situation, the availability of males could mean maximizing fertility or longevity for females, which could assure further mating opportunities.
